# Clinical Course and Dedifferentiation of Atypical Lipomatous Tumors: A Retrospective Analysis of Surveillance and Surgical Management

**DOI:** 10.1245/s10434-026-19751-6

**Published:** 2026-05-13

**Authors:** S. N. Hakkesteegt, J. Shapiro, J. S. Wunder, K. M. Tsoi, A. M. Griffin, L. M. White, R. A. Gladdy, C. Verhoef, D. J. Grunhagen, P. C. Ferguson

**Affiliations:** 1 Department of Surgical Oncology and Gastrointestinal Surgery, Erasmus MC Cancer Institute Erasmus University Medical Center, Rotterdam, The Netherlands; 2 Department of Surgical Oncology, Radboud University Medical Center, Nijmegen, The Netherlands; 3 Department of Surgery, University of Toronto, Toronto, Canada; 4 Division of Orthopedic Surgery, Mount Sinai Hospital, University Musculoskeletal Oncology Unit, Toronto, ON Canada; 5 Joint Department of Medical Imaging, Mount Sinai Hospital and Women’s College Hospital, University Health Network, Toronto, Canada; 6 Department of Medical Imaging, Temerty Faculty of Medicine, University of Toronto, Toronto, Canada; 7 Institute of Medical Science, University of Toronto, Toronto, Canada; 8 Lunenfeld-Tanenbaum Research Institute, Mount Sinai Hospital, Toronto, Canada

**Keywords:** Atypical lipomatous tumor, Surveillance, Dedifferentiated liposarcoma, Resection, Liposarcoma

## Abstract

**Background:**

Atypical lipomatous tumors (ALTs) can recur locally but do not metastasize. Surgical treatment risks morbidity, recurrence, and dedifferentiation. This study assessed the outcomes of initial surveillance or surgery for patients with ALT.

**Methods:**

Patients with ALT diagnosed between 2005 and 2022 were retrospectively identified from surgical and radiologic databases. Adults with primary or locally recurrent (LR) ALT confirmed by imaging, histology, or both and managed with surveillance for 12 months or longer were included in the surveillance study cohort. The primary outcome was the development of dedifferentiation during surveillance. The secondary outcomes were tumor progression, imaging-histopathology concordance, motives for surgery, and surgical outcomes. A comparison cohort included patients who had ALT treated with upfront surgery.

**Results:**

The 36 patients (median age, 63 years; IQR, 52–69 years) in this study underwent surveillance for a median of 26 months (IQR, 19–54 months). Most of the tumors were primary (81%) and located in the lower extremities (78%). Tumor progression (RECIST) occurred in 22 patients (61%). Three patients (8%) experienced dedifferentiation (2 with the diagnosis by imaging alone at baseline and 1 with an LR). No dedifferentiation occurred in the remaining 33 patients (92%). Magnetic resonance imaging suggested dedifferentiation in five cases, histologically confirmed in two cases. Among 147 patients initially treated surgically for ALT, 15 (10%) had LR, with 3 (20%) of these patients experiencing recurrence as dedifferentiated liposarcoma. No disease-related metastases or deaths occurred.

**Conclusion:**

Initial surveillance for ALT appears feasible for selected patients. However, surgery remains the preferred treatment for many, and the short follow-up period and small cohort size limited definitive conclusions regarding long-term dedifferentiation risk. Histologic confirmation remains essential when MRI findings are atypical.

Atypical lipomatous tumors (ALTs) are low-grade soft tissue tumors characterized by amplification of the Murine double minute 2 (MDM2) oncogene.^1^ The prognosis for patients with a diagnosis of ALT is excellent, with reported survival rates ranging from 90 to 100% after 5 to 10 years of follow-up evaluation.^2–6^ As a result, ALTs are considered intermediate (locally aggressive) tumors that can recur locally after resection but do not metastasize.^1,7,8^

The terminology used for these tumors depends on their anatomic location. When located in deep regions such as the retroperitoneum, mediastinum, or spermatic cord, they are referred to as well-differentiated liposarcomas (WDLPS) despite being biologically identical to peripherally located ALTs. Both ALTs and WDLPS carry the potential to transform into dedifferentiated liposarcoma (DDLPS), which is associated with a significantly worse prognosis. This risk is minimal for ALTs (< 4%),^2,3,9–11^ but notably higher for WDLPSs (> 20 %)^12,13^ primarily due to the increased likelihood of positive resection margins and tumor recurrence in deep-seated locations.

Interestingly, previous studies have shown that dedifferentiation in ALTs has been observed only in locally recurrent tumors after initial surgical treatment.^3,14^ However, it remains unclear whether dedifferentiation is a time-dependent process driven by underlying genetic factors or a condition triggered by surgical intervention.^12,15^

Despite the excellent prognosis, many patients with an ALT undergo extensive surgical procedures, risking complications and inducing morbidity.^6,14^ Although active surveillance has become an accepted approach for managing other low-grade intermediate tumors,^16^ it is less commonly used as a primary strategy for ALTs. The active surveillance approach in ALTs may be attractive because these tumors tend to grow slowly, are commonly asymptomatic, can require extensive surgery when ill-located, and have relatively high recurrence rates.

Previous studies have suggested that active surveillance may be a viable option for selected patients with an ALT.^14^ Building on this evidence, the MINIMALIST trial (NL72207.078.20) was initiated to prospectively evaluate the feasibility of surveillance for patients with a diagnosis of ALT, with a focus on patient preferences, health-related quality of life, and natural tumor behavior. However, aside from these limited studies, the outcomes of surveillance for ALTs are rarely reported in the current literature. Given the rarity of dedifferentiation and the long interval during which it may occur, robust data remain limited.

This study aimed to evaluate the natural tumor behavior of ALTs during surveillance, especially the development of dedifferentiation, in patients monitored in a single large sarcoma expertise center.

## Methods

Patients with a diagnosis of ALT who were initially monitored for a minimum of 12 months during 2005–2022 were identified from a prospectively maintained surgical database at Mount Sinai Hospital (MSH), Toronto, Canada. Additionally, patients not initially registered in the surgical database because of an initial nonsurgical approach were separately identified through the Medical Imaging Department at MSH by a search of MR imaging records for the terms “atypical lipomatous tumor,” “ALT,” and “well-differentiated liposarcoma.” This approach captured only patients who underwent imaging at MSH. Patients whose imaging was performed at referring centers or through their general practitioner could not be identified using this method.

The inclusion criteria specified adult patients with a primary or locally recurrent (LR) ALT who were monitored with MR imaging for a minimum of 12 months. The diagnosis of ALT was based primarily on characteristic MRI features, and histologic confirmation was obtained either with core needle biopsy (CNB) or after surgical resection. All included cases were ultimately confirmed histologically.

The exclusion criteria ruled out MDM2-negative tumors tested with fluorescence *in situ* hybridization (FISH); polymerase chain reaction (PCR) or immunostaining; tumor location in the mediastinum, retroperitoneum, or testis/scrotum (as these are considered WDLPS); a monitoring period shorter than 12 months; and no available imaging during follow-up evaluation. Patients who had surgical resection of an ALT with no preceding monitoring or a monitoring period shorter than 12 months were categorized as “initially treated with surgery” and analyzed and described separately.

The primary study objective was to assess the occurrence of dedifferentiation during surveillance. The secondary study objectives included evaluating tumor progression during surveillance, motives for switching to surgery, agreement between imaging and histopathology when dedifferentiation was suspected, and surgical outcomes (either as initial treatment or after a monitoring period of at least 12 months). Multiple patient, tumor, and treatment-related characteristics were collected. These included age, gender, comorbidities, primary or recurrent tumor, tumor size (at presentation and during monitoring), site, symptoms, pathology report including MDM2 amplification status, (neo) adjuvant treatment and surgical details, occurrence of metastases, and disease-related survival. For patients with recurrent tumors, treatment history and time to recurrence also were documented.

Imaging data included tumor size in three dimensions and signs of dedifferentiation determined by a musculoskeletal radiologist. Progressive disease was defined according to RECIST criteria^17^ on two consecutive MR scans. Dedifferentiation was assessed on MR imaging based on tumor morphology, signal characteristics, and contrast enhancement patterns (i.e., nodular, non-fatty components adjacent to fatty components are highly suggestive for dedifferentiation).^18–20^ For patients described in radiology reports as having experienced dedifferentiation, MR images were re-reviewed by a musculoskeletal radiologist to confirm the finding. Dedifferentiation had to be proven histologically upon biopsy or the resected specimen.

### Statistical Analysis

This study primarily involved descriptive data analysis. Continuous variables were summarized using medians with interquartile ranges (IQRs). Categorical variables were presented as counts and percentages. Data were compiled and analyzed using R statistical programming version 4.0.3 to ensure accurate and consistent reporting. No inferential statistical tests were performed because the study design did not include group comparisons or hypothesis testing.

## Results

**Fig. 1:**
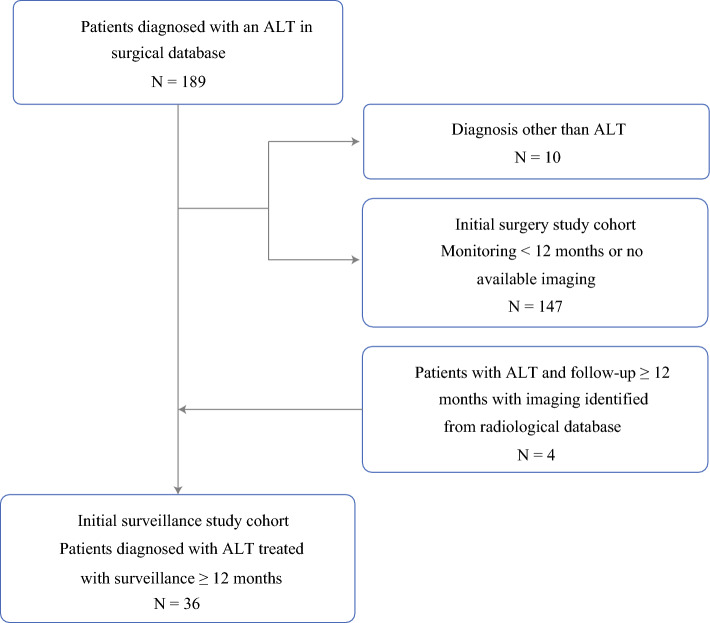
Flow diagram illustrating the selection of patients into the surveillance cohort and the surgery cohort

The study identified 189 patients with an initial diagnosis of ALT from a prospectively maintained surgical database. Of these patients, 10 were excluded after histopathologic review confirmed a diagnosis other than ALT. From the surveillance study cohort, 147 patients with resected ALTs were excluded due to either lack of available imaging or a monitoring period shorter than 12 months before lesion excision and comprised the upfront surgical cohort (Fig. 1). Four additional patients meeting the inclusion criteria were identified through a radiologic database search. This resulted in a final group of 36 patients in the surveillance cohort (Fig. 1), which had a balanced male-to-female ratio and a median age of 63 years (IQR, 52–69 years) at first presentation.

Most ALTs in the surveillance cohort were primary tumors (81%) located in the lower extremity (78%), with a median tumor size of 14.8 cm (IQR, 10.1–19.6 cm) at presentation (Table 1). Diagnosis of ALT was established based on MRI findings and later confirmed histologically on the resected specimen for 25 patients (69%), by percutaneous CNB for 6 patients (17%), or by residual/recurrent disease diagnosed after prior surgical resection of a histopathologically confirmed ALT for 5 patients (14%).

**Table 1:** Characteristics of the patients initially treated with surveillance or upfront surgery

	Surveillance (*n* = 36) *n* (%)	Upfront surgery (*n* = 147) *n* (%)
Gender		
Female Male	18 (50) 18 (50)	62 (42) 85 (58)
Median age at presentation: years (IQR)	62.5 (52–69)	60.0 (51–69)
Tumor location		
Upper extremity Lower extremity Chest wall Paraspinal	5 (14) 28 (78) 2 (5) 1 (3)	20 (14) 121 (82) 3 (2) 3 (2)
Diagnosis through FISH^a^	30 (83)	43 (29)
Presenting status		
Primary tumor Recurrent tumor	29 (81) 7 (19)	130 (88) 17 (12)
Median duration of surveillance: months (IQR)	26 (19–54)	NA
Median tumor size at intake: cm (IQR)	14.8 (10.1–19.6)	16.7 (11.1–21.4)
Tumor growth during surveillance		
Any progression RECIST progression Median tumor growth: cm (IQR)	31 (86) 22 (61) 3.6 (1.8–6.0)	NA NA NA
Dedifferentiation during surveillance^b^		
MRI features suggestive of dedifferentiation Dedifferentiation confirmed in the resected specimen	5 (14) 3 (8)	NA NA
Surgery during follow-up	32 (89)	NA
(Neo)adjuvant treatment		
Neoadjuvant radiotherapy^c^ Adjuvant radiotherapy	2 (6) 1 (3)	7 (5) 0 (0)
Surgical complications^d^	1 (3)	12 (8)
Distant metastases and/or disease related death	0 (0)	0 (0)

IQR, interquartile range; FISH, fluorescence *in situ* hybridization; NA, not applicable; RECIST, Response Evaluation Criteria In Solid Tumors

^a^The remainder was diagnosed through immunohistochemistry alone or reverse transcription polymerase chain reaction (RT-PCR)

^b^Of the five patients with radiologic suspicion of dedifferentiation, two were histologically proven. One other patient did not have radiologic suspicion of dedifferentiation, but areas with low-grade dedifferentiation were found in the resected specimen

^c^Both cases had MRI features suggestive of dedifferentiation; in one patient this was histologically proven

^d^A patient who experienced dedifferentiation during the follow-up period (patient 2, Table 2). The patient did not receive any radiation therapy but suffered from multiple complications requiring prolonged hospital stay and vacuum-assisted wound closure (VAC)

All the patients in the surveillance cohort had an intake MRI exam and at least one additional follow-up MR imaging study available during surveillance. During a median surveillance length of 26 months (IQR, 19–54 months) (Table 1), the patients underwent a median of two follow-up MRIs (IQR, 2–3).

### Tumor Dedifferentiation and Tumor Progression During Surveillance

In 3 (8%) of the 36 patients in the surveillance cohort, dedifferentiation was observed in the resection specimen after initial ALT diagnosis. (Table 2). In one patient, dedifferentiation occurred 19 years after the initial ALT diagnosis and 7 years after a local recurrence (LR) of the resected ALT had developed (Table 2, patient 3). In the remaining two patients, the ALT was diagnosed radiologically without histologic confirmation before initiation of surveillance. In one of these two patients, features suggestive of dedifferentiation were not present on the initial MR imaging examination, but were observed on a follow-up MR imaging exam performed 47 months later. Features of dedifferentiation in this tumor were histopathologically proven with a preoperative image-guided CNB and confirmed again on pathologic evaluation of the resected specimen (Table 2, patient 2). The third patient had no signs suspicious for dedifferentiation on initial MR imaging, but this patient proceeded to surgery 9 years later due to symptoms, and the resected specimen showed areas of low-grade dedifferentiation on histologic assessment (Table 2, patient 1).

**Table 2:** Patients with an ALT who underwent initial surveillance and were later found to have dedifferentiation upon resection

Primary tumor						Local recurrence		Dedifferentiation			Monitoring period (months)	Metastases or disease-related mortality
No.	Age (years)	Year	Location	Diagnosis	Treatment	Year	Treatment	Detection	Surgery	RTx		
**1**	58	2010	Lower extremity (DS)	MRI	Monitoring	NA	NA	None^a^	2019	Postop	111	No
**2**	78	2010	Lower extremity (DS)	MRI	Monitoring	NA	NA	CNB	2015	No^b^	47	No
**3**	45	2005	Upper extremity (DS)	MRI, resected specimen	Surgery: microscopic positive margins^c^	2017	Monitoring	CNB	2024	Preop	83	No

ALT, atypical lipomatous tumor; RTx, radiotherapy; MRI, magnetic resonance imaging; NA, not applicable; DS, deep-seated; CNB, core needle biopsy

^a^The patient had ALT diagnosed based on MR findings and eventually underwent surgery due to development of symptoms. Imaging showed no signs of dedifferentiation. However, postoperative histopathologic examination showed areas of low-grade dedifferentiation

^b^The surgical margins were positive with ALT. Due to multiple comorbidities, the patient did not receive any radiotherapy

^c^The tumor encased the brachial plexus, and the patient underwent multiple surgical procedures due to residual or recurrent disease. Monitoring was initiated in 2017 and continued until the development of dedifferentiated liposarcoma (DDLPS) in 2024, when preoperative radiation was performed followed by surgical resection

Across the remaining 33 surveillance patients (92%), 29 eventually underwent surgery due to development of symptoms, and none had histologic findings of dedifferentiation. The remaining four patients not resected during surveillance also had no MR imaging features concerning for dedifferentiation. No metastases or disease-related deaths occurred.

During surveillance, RECIST progressive disease (PD) was observed in 22 patients (61%). Any progression (RECIST PD or tumor growth  < 20%) was identified in 31 (86%) patients during the study period (Table 1).

### Suspicion of Dedifferentiation on MRI and Histologic Concordance

During surveillance, five patients (14%) experienced radiologic signs suspicious of dedifferentiation on MRI that led to surgery. In two of these five patients, dedifferentiation was proven preoperatively with CNB, and they were subsequently treated surgically (Table 2, patients 2 and 3). In the remaining three patients, there was a high suspicion for dedifferentiation based on MR imaging. These three patients underwent resection without preoperative CNB, but no dedifferentiation was detected histopathologically in the resected specimens.

### Transition from Monitoring to Surgery

Most of the patients (32 of 36, 89%) eventually underwent surgery after an initial period of monitoring, at a median of 36 months (IQR, 22–56 months). In 5 (14%) of these 36 patients, follow-up surveillance showed MRI features suspicious for dedifferentiation on imaging that led to surgery. These were observed during a median interval of 19 months (IQR, 13–62 months) after initiation of tumor surveillance. In the remaining 27 patients (75%) proceeding to tumor resection, the main reasons for surgery were development of tumor-related discomfort, functional distress, anxiety, cosmetic considerations, or physician’s preference (Table 1).

### Patients Initially Treated With Surgery

Upfront surgery was performed for 147 patients who had no initial surveillance period with imaging (or surveillance shorter than 12 months) before surgery. Of these patients, 15 (10%) experienced a local recurrence at a median of 63 months (IQR, 55–109 months) postoperatively. These patients were not routinely monitored postoperatively, but presented with a recurrent swelling or symptoms at the surgical site. The local re-recurrence rate was 33% (5 of 15 recurrences), with five patients experiencing a second (*n* = 2), third (*n* = 2), or fourth (*n* = 1) LR after repeated surgery. Of these 15 patients with an LR, 3 (20%) experienced dedifferentiated liposarcoma that required surgery and radiotherapy. One patient experienced a second dedifferentiated LR 2 years later and was successfully re-treated. No metastases or disease-related deaths occurred for any of these patients.

## Discussion

This study evaluated the natural tumor behavior of ALTs in patients undergoing initial surveillance and demonstrated dedifferentiation in only 8% of patients during a median follow-up period of 26 months. Two (8%) of the three patients who did have dedifferentiation in the resected specimen initially had ALT diagnosed on MRI. Because the initial ALT diagnosis for these two patients was not confirmed on histopathology, the presence of dedifferentiated areas within the ALT at first presentation cannot be entirely excluded. The third patient experienced dedifferentiation after presenting with a LR. Our findings align with previous literature,^3,11,14,21^ suggesting that predominantly recurrent ALTs carry a risk of dedifferentiation, whereas this risk is negligible in primary ALTs but can rarely develop over time if the tumor is not resected.

Currently, the gold standard for diagnosing ALTs is histopathologic evaluation combined with FISH or reverse transcription (RT)-PCR to detect MDM2 gene amplification, although some centers rely on MRI evaluation alone. With the increased use of CNB in which only limited tissue is available for histologic evaluation, the utilization of molecular testing for MDM2 gene amplification provides a highly sensitive and specific method for diagnosing ALTs.^22^ A recent study evaluated the accuracy of CNB and FISH in diagnosing adipocytic tumors and reported a 95% concordance between CNB findings and the final diagnosis after surgical excision.^23^ Despite the high diagnostic accuracy, cases have been described in which ALTs were upgraded to DDLPS after resection, likely due to tumor heterogeneity and the potential for CNB sampling error.^23,24^ Alternative diagnostic approaches for ALT, primarily involving imaging techniques, also have been explored. In the literature, various imaging features have been identified to aid in the MRI-based diagnosis of ALTs, including tumor size, tumor depth, and the presence of thick/enhancing septations.^25–28^ However, studies comparing the accuracy of these imaging features with that of histopathology are lacking. Given the high sensitivity and specificity of FISH, together with the strong diagnostic accuracy of CNB, histopathologic confirmation is more accurate than MRI imaging alone and should be used for cases in which MRI findings are atypical or concerning for dedifferentiation before initiation of surveillance.

In this study, three patients had MRI features suggestive of dedifferentiation during surveillance, which was not confirmed histologically or molecularly in the resected specimens. For asymptomatic patients who opt for surveillance of ALT, image-guided CNB targeting non-fatty components of the tumor can help avoid the need for surgery by ruling out radiologically suspected dedifferentiation. However, the lower sensitivity and specificity of CNB in diagnosing DDLPS should be carefully considered,^23,24,29^ and surgical resection can be justified when MRI findings raise suspicion for dedifferentiation, even in the presence of a negative biopsy.

Because ALTs are considered intermediate tumors that do not metastasize and very rarely dedifferentiate as also presented in our study, it is debated whether surgery should still be the standard of care. Surgical treatment consisting of marginal resection is the accepted therapy for ALTs. Although higher local recurrence rates have been reported compared with wide resection, the morbidity and complication risks are lower and there is no difference in overall survival.^21,30,31^ Aligning with previous literature,^3,11,14,21^ we observed an LR rate of 10% (15/147 cases) for the patients who underwent upfront surgery for ALT. Of these 15 patients, 3 (20%) experienced a dedifferentiated LR requiring extensive oncologic treatment due to the metastatic potential of DDLPS. For this reason, Vos et al.^14^ suggested that we might be overtreating these patients and that surveillance may be a feasible alternative for selected patients, especially those who are minimally symptomatic or asymptomatic, thereby preventing surgery-induced morbidity, mortality, and dedifferentiated local recurrences. In their study, 24 (12.6%) of 191 patients with an ALT underwent surveillance, 4 (16.7%) of whom eventually had surgical resection at a median of 1.8 years due to tumor growth or symptoms, compared with 36 (19.7%) of 183 patients in our study. Notably, none of the primary ALTs resected after initial surveillance in either study showed evidence of dedifferentiation. Based on the previous literature and the current study, an initial nonoperative management seems safe and can be considered a viable alternative based on patient and tumor characteristics and the patient’s preference.

In contrast to the patients managed with surveillance, who were largely asymptomatic or minimally symptomatic, most of the patients undergoing upfront surgery presented with symptoms, tumor growth, or both and therefore opted for surgical intervention. Importantly, the choice for upfront resection is not solely driven by objective tumor behavior. Some patients with minimal symptoms or limited tumor growth may still prefer surgical treatment. Such preferences may reflect prior personal or familial experiences with cancer, anxiety regarding future tumor progression, or concern that delayed surgery could result in a larger and more complex procedure with increased risk of postoperative complications or functional limitations. These considerations underscore the importance of individualized counseling that integrates clinical factors with patient values and expectations. Moreover, surgery remains the preferred therapy for patients with symptoms, histologic evidence of dedifferentiation on biopsy, or concern for dedifferentiation on MRI.

Currently, no formal guidelines exist regarding initial surveillance and postoperative follow-up evaluation for patients with an ALT. Risk of dedifferentiation in our surveillance group was low and occurred only late after LR or after many years of follow-up evaluation, and this low risk is supported by the study of Vos et al.^14^ Therefore, a further de-escalation of surveillance is applied in our clinic. Patients with a primary ALT who do not want surgical treatment are not always routinely monitored with MRI or scheduled outpatient clinic visits, but are instructed to contact the clinic in the event of new symptoms or suspected tumor growth, at which point additional clinical assessment and imaging can be performed. In that way, the burden of scheduled MRIs and associated health care costs can be reduced. Furthermore, if patients are surgically treated, they do not routinely undergo further follow-up visits or imaging at our center. Instead, they are instructed to contact the outpatient clinic in the event of recurrent symptoms or concern for LR. Only in such cases are additional clinical evaluation and imaging performed. However, available data remain insufficient to support definitive recommendations. Identifying those at risk of dedifferentiation (i.e., patients with an LR) could enable more personalized management strategies while sparing others from unnecessary surgeries, imaging, and hospital visits. Future studies on genomic changes associated with progression from ALT to DDLPS as well as prospective multicenter studies including assessment of health-related quality of life (HrQoL) during surveillance (i.e., the MINIMALIST trial; NL72207.078.20) are anticipated to offer new helpful insights.

This study had a number of limitations. First, a retrospective dataset was used including patients who underwent surgical treatment at some point, introducing bias. Data from patients who did not undergo surgery were not routinely collected. Therefore, the current study cohort underestimated the true number of patients managed with surveillance during the study period by a large degree. Although the hospital radiology database also was queried in an attempt to include more patients in the surveillance group who were not captured in the surgical database and never had surgery, most of these patients had their MRI scans performed elsewhere before referral and therefore could not be identified and probably represented the majority of surveillance cases. Hence the proportion of patients who undergo surgery for ALT after surveillance and the proportion who experience dedifferentiation are likely to be significantly less than reported in this study. In addition, for most of the patients, a biopsy was not performed before surveillance was initiated, and the diagnosis of ALT was made based on MRI findings. Because two patients without a pre-monitoring CNB had DDLPS diagnosed following resection after initial prolonged surveillance, it remains uncertain whether these cases represent true dedifferentiation during surveillance or whether DDLPS would have been identified had CNB been initially performed, although in neither case was there any radiologic concern for DDLPS. Furthermore, the observed 8% dedifferentiation rate in the surveillance cohort was based on very small numbers and may reflect selection bias, incomplete baseline histologic assessment, or sampling limitations rather than true biologic progression. Yet, based on the aforementioned limitations in identifying all the patients with ALT who did not undergo surgery, the dedifferentiation risk is likely to be much lower. Finally, the relatively short follow-up duration was insufficient to assess the true rate of late dedifferentiation and prevented us from drawing conclusions about the long-term behavior of ALTs. Therefore, our findings should not be interpreted as evidence that surveillance confers a low long-term risk.

In conclusion, surveillance may be a feasible initial management strategy for selected patients with ALT, particularly when symptoms are minimal. However, surgery remains the preferred therapy for a substantial proportion of patients, and the relative short follow-up period and small surveillance cohort preclude firm conclusions regarding long-term dedifferentiation risk. It seems reasonable to identify patients with an ALT for initial surveillance based on MRI findings, recognizing that CNB and molecular testing are more accurate and should be performed whenever the MRI is atypical or concerning for dedifferentiation. Larger, prospective trials with longer follow-up periods are expected to provide further insight into the feasibility and safety of surveillance, long-term tumor behavior, and HrQoL for patients with an ALT.
